# Long non-coding RNA XIST regulates PTEN expression by sponging miR-181a and promotes hepatocellular carcinoma progression

**DOI:** 10.1186/s12885-017-3216-6

**Published:** 2017-04-07

**Authors:** Shuzhen Chang, Binhe Chen, Xiaoyan Wang, Keqin Wu, Yuqiu Sun

**Affiliations:** 1 Division of Liver Disease, Ji’nan Infectious Disease Hospital, No. 22029 Jingshi Road, Ji’nan, Shandong 250021 China; 2 Healthy Food Laboratory, Shandong Academy of Pharmaceutical Sciences, Ji’nan, Shandong 250101 China

**Keywords:** MicroRNA-181a, Hepatocellular carcinoma (HCC), Metastasis, PTEN, XIST

## Abstract

**Background:**

Tumor metastasis often occurs in hepatocellular carcinoma (HCC) and influences the patient’s prognosis, and microRNAs are reported to play key roles in tumor metastasis. This study was conducted to explore the effect of microRNAs on HCC metastasis.

**Methods:**

The levels of miR-181a in HCC tissues, adjacent tissues, metastatic HCC tissues, and non-metastatic HCC tissues at different stages were determined by qRT-PCR. Effect of miR-181a on the proliferation, invasion, and metastasis of HCC cells was estimated by cell counting kits-8 (CCK-8), wound-healing, and Transwell assays. Software analysis and luciferase assays were used to explore the target gene of miR-181a.

**Results:**

MiR-181a was up-regulated in HCC tissues and its expression level in metastatic HCC tissues was much higher than in non-metastasis samples. PTEN was found to be a target gene of miR-181a. MiR-181a had multiple binding sites with the long non-coding RNA (lncRNA) XIST. The regulation of miR-181a on PTEN was mediated by lncRNA XIST. The proliferation and invasion of cells with siXIST were significantly enhanced compared with those of control cells, while knockdown of miR-181a abolished the enhancing effects.

**Conclusions:**

MiR-181a can promote HCC metastasis by targeting PTEN, which is regulated by lncRNA XIST.

**Electronic supplementary material:**

The online version of this article (doi:10.1186/s12885-017-3216-6) contains supplementary material, which is available to authorized users.

## Background

Hepatocellular carcinoma (HCC) is one of the most ubiquitous tumors in the world [1]. It grows rapidly and has a high propensity for metastasis, which worsens the prognosis of patients with this neoplasm [2]. Therefore, HCC metastasis is a significant problem that needs to be addressed [3]. However, the molecular mechanisms by which HCC metastasis occurs are still unknown and need to be elucidated.

Epithelial-to-mesenchymal transition (EMT) plays an important role in regulating metastasis and invasion by malignant tumors [4], including HCC [5]. Many factors have been reported to influence EMT, including signaling pathways and microRNAs. Moreover, previous studies showed that abnormal expression of some microRNAs (miRNAs) were related with the development of HCC [6]. Philip et al. [7] reported that the miR-200 family and miR-205 can regulate EMT by targeting ZEB1 and SIP1 (SIP1, also named ZEB-2, is a smad-interacting, multi-zinc finger protein that shows specific DNA binding activity). Adam et al. [8] reported members of the miR-200 family appear to control the EMT process in bladder cancer cells. Zhang et al. [9] reported miR-27 plays an important role in regulating metastasis of gastric cancer by inducing EMT. In hepatocytes, miR-30 can inhibit TGF-β1-induced EMT by targeting Snail1 (Snail1 is a zinc finger transcriptional repressor whose pathological expression has been linked to cancer cell) [10]. Moreover, in HCC, miR-490-3p can modulate cell growth and EMT of HCC cells by targeting endoplasmic reticulum-golgi intermediate compartment protein 3 (ERGIC3) [11]. However, the changes in miRNA expression in response to EMT are highly complex and variable.

In the present study, we used Hsa-miR-181a-5p, which is a mature product of hsa-miR-181a with a sequence of 24-AACAUUCAACGCUGUCGGUGAGU-46. We found that miR-181a was up-regulated in the tumor tissues of HCC patients and promoted the proliferation and metastasis of HCC cells in vitro and in vivo. We investigated the molecular mechanism of miR-181a in the progression of HCC and the reciprocal regulation between miR-181a and the long non-coding RNA (lncRNA) XIST.

## Methods

### Identification of differentially expressed miRNA

The gene expression profiles of HCC patients with normal controls were downloaded from Gene Expression Omnibus (http://www.ncbi.nlm.nih.gov/geo) with accession number of GSE77314. This dataset was based on the platform of IlluminahumanHT-12 v4.0 expression beadchip deposited by Seok et al. [12]. In their study, Seok et al. focused on comparing molecular features of scirrhous hepatocellular carcinoma with those of hepatocellular carcinoma and CC. The original dataset included 26 genechips, and we extracted 11 of them for further analysis, including 6 from CC cancer tissue and 5 from the surrounding normal liver.

We transferred the probe-level data in CEL files into expression measures. Then, background was corrected and quartile data was normalized by the robust multi-array average (RMA) algorithm. The file in the platform annotation files provided by the Affymetrix Company was used to map the relationship between the probes and gene symbols. A probe would be filtered if it did not have corresponding gene symbols. The average value of gene symbols with multiple probes obtained was then further analyzed. A set of gene-specific t-tests with the threshold of false discovery rate (FDR) ≤ 0.05 was assimilated to identify differentially expressed miRNA.

### Tissue samples

We collected both cancer tissue and adjacent normal tissue from 55 HCC patients who underwent surgery in the Ji’nan Infectious Disease Hospital between February 2013 and November 2015. Before the surgery, they had no chemotherapy or radiotherapy performed. After resection, the tissues were stored at −80 °C. The stages of tissues were distinguished based on the seventh edition of the American Joint Committee on Cancer (AJCC) tumor node-metastasis (TNM) staging system.

This study was approved by the Ethics Committees of Ji’nan Infectious Disease Hospital. Informed consent was obtained from each patient, including consent for their samples to be taken and used for research purposes.

### Cell culture

Human HCC cell lines HCCLM3, HepG2, Hep3B, SMMC-7721, and Huh7 as well as normal liver cell lines HL-7702 and L-02 were purchased from the Cell Bank of the Chinese Academy of Sciences (Shanghai, China). All cells were kept in Dulbecco’s Modified Eagle’s Medium (DMEM, Gibco, USA) in humidified air containing 5% CO_2_ at 37 °C. This medium contained 10% fetal bovine serum (FBS) (Hyclone, USA) and 1% penicillin/streptomycin.

### Cell transfection

Cells were seeded into 6-well plates and transfected with miR-181a mimics, miR-181a inhibitor, or normal control (NC) using Lipofectamine 2000 (Invitrogen, USA). For inhibiting endogenous lncRNA XIST expression, siRNA targeting XIST was purchased from Shanghai GenePharma, China. The partial sequences used in this study are shown in Additional file 1: Table S1.

### RNA isolation and quantitative RT-PCR

RNA of microRNA used for detection was extracted from tissues or cell lines using a mirVana miRNA isolation kit. RNA of mRNA used for detection was extracted from tissues or cell lines using the TRIzol method. TRIzol (1 mL) was added and the solution was mixed for 10 min until it was homogeneous. The mixture was then transferred into Eppendorf tubes (EP, 1.5 mL) with 200 μl chloroform. After shaking for 15 min, the EP tubes were centrifuged at 4 °C for 15 min (12,000×g). The supernatant was transferred into other EP tubes and mixed with isopycnic isopropanol for 15 s. The mixture was then centrifuged at 4 °C for 10 min (12,000×g). The supernatant was discarded and the precipitate was washed with 75% ethanol twice and dissolved into 30 μl diethylpyrocarbonate (DEPC) after drying to obtain an RNA stock solution. After isolation, the concentration of RNA was determined using a NanoDrop 1000 spectrophotometer (NanoDrop Technologies, Wilmington, Delaware, USA), and the RNA solution was stored at −80 °C for further use.

For qRT-PCR, genes were amplified by specific oligonucleotide primers, and the human glyceraldehyde-3-phosphate dehydrogenase (GAPDH) gene was used as an endogenous control. The sequences of primers and probes used for the qRT-PCR analysis are shown in Additional file 2: Table S2. The detection and quantification entailed the following steps: (1) reverse transcription was performed at 55 °C for 30 min, (2) initial activation for 15 min at 95 °C, (3) 40 cycles of denaturation conducted at 94 °C for 15 s, (4) annealing for 30 s at 55 °C, and (5) extension for 30 s at 72 °C. The expression level was normalized using U6 small nuclear RNA by the 2^−ΔCt^ method.

### Western blotting

Cells were inoculated to 6-well plates with each plate of 5 × 10^5^ cells and cultivated for 24 h. The culture solution was absorbed away and cells were rinsed with ice PBS for 3 times. Then radio immunoprecipitation assay (RIPA) was added to each well to obtain total proteins; 12% separation gel and 5% spacer gel was then used to perform SDS-PAGE gel electrophoresis. Samples were transferred to nitrocellulose filter membranes (Hybond, Escondido, CA, USA). The membranes was placed in tris-buffered saline Tween-20 (TBST) containing 5% skim milk powder and blocked for 1 h at room temperature. After 1 h, membranes were taken out and rinsed with TBST once. Antibodies were diluted with 5% bovine serum albumin (BSA), and membranes were placed in antibody with the appropriate concentration. Primary antibodies were determined using an EZ-ECL chemiluminescence Detection kit for HRP (Biological Industries, Beit-Haemek, Israel).

### Cell viability assay

Cells were seeded in the 96-well plate for 24 h after transfection at a density of 1500 cells/well. The cell viability assay was performed using a Cell Counting Kit-8 (CCK8; Dojindo) according to the manufacturer’s protocol. The absorbance at 450 nm was measured. Experiments were performed in triplicate.

### Wound healing assay

After transfection of miR-181a mimics, miR-181a inhibitor or NC, HCCLM3 and Huh7 cells (1 × 10^6^/well) were inoculated to 6-well plates. Wounds were imposed by dragging a 1000-μl pipette tip through the cell monolayer. Cells were allowed to migrate for 36 h. The gap area was then photographed, and migration distances were measured.

### Cell invasion assay

Invasion activity of the cells was determined with a cell invasion assay according to the manufacturer’s instructions (BD Biosciences, San Jose, CA, USA). Briefly, HCCLM3 and Huh7 cells (5 × 10^4^/well) were seeded in the upper chamber insert with 1% FBS medium after transfection, while the lower chamber insert contained complete culture medium with 10% FBS medium to trap invading cells. After incubation for 24 h, cells that penetrated the Matrigel-coated membranes and migrated into the lower chamber were stained with crystal violet (0.1%) and photographed. In each sample, invasion ability was quantified by counting crystal violet-stained cells.

### Luciferase activity assays

The 3′-UTR of PTEN sequence was cloned into the pGL3-basic luciferase reporter vector (Promega, USA). For the luciferase assays, 100 ng PGL3-PTEN-3’UTR vector was co-transfected in cells with 100 nM miR-181a mimics or control regent, together with 20 ng Renilla luciferase vector (Promega, USA) as an internal normalized control. Cells were harvested after transfection for 48 h, and their luciferase activities were determined according to the manufacturer’s protocol. Transfections were performed in duplicate and repeated three times.

### Tumor size and weight

After HCC cell lines were transfected with miR-181a mimics or miR-181a inhibitor for 1, 2, 3, 4, or 5 days, we determined the tumor size and tumor weight, and compared them with those of the controls. The tumor size was determined by three-dimensional ultrasound, and tumor weight was determined by a precision electronic balance.

### Statistical analysis

Statistical analyses were performed using SPSS (SPSS Inc., Chicago, IL, USA). All data were expressed as mean ± standard deviation. Student’s t-test or a one-way ANOVA test was performed to determine significant differences; a *p < 0.05* was considered statistically significant.

## Results

### MiR-181a was up-regulated in HCC metastasis patients

**Fig. 1:**
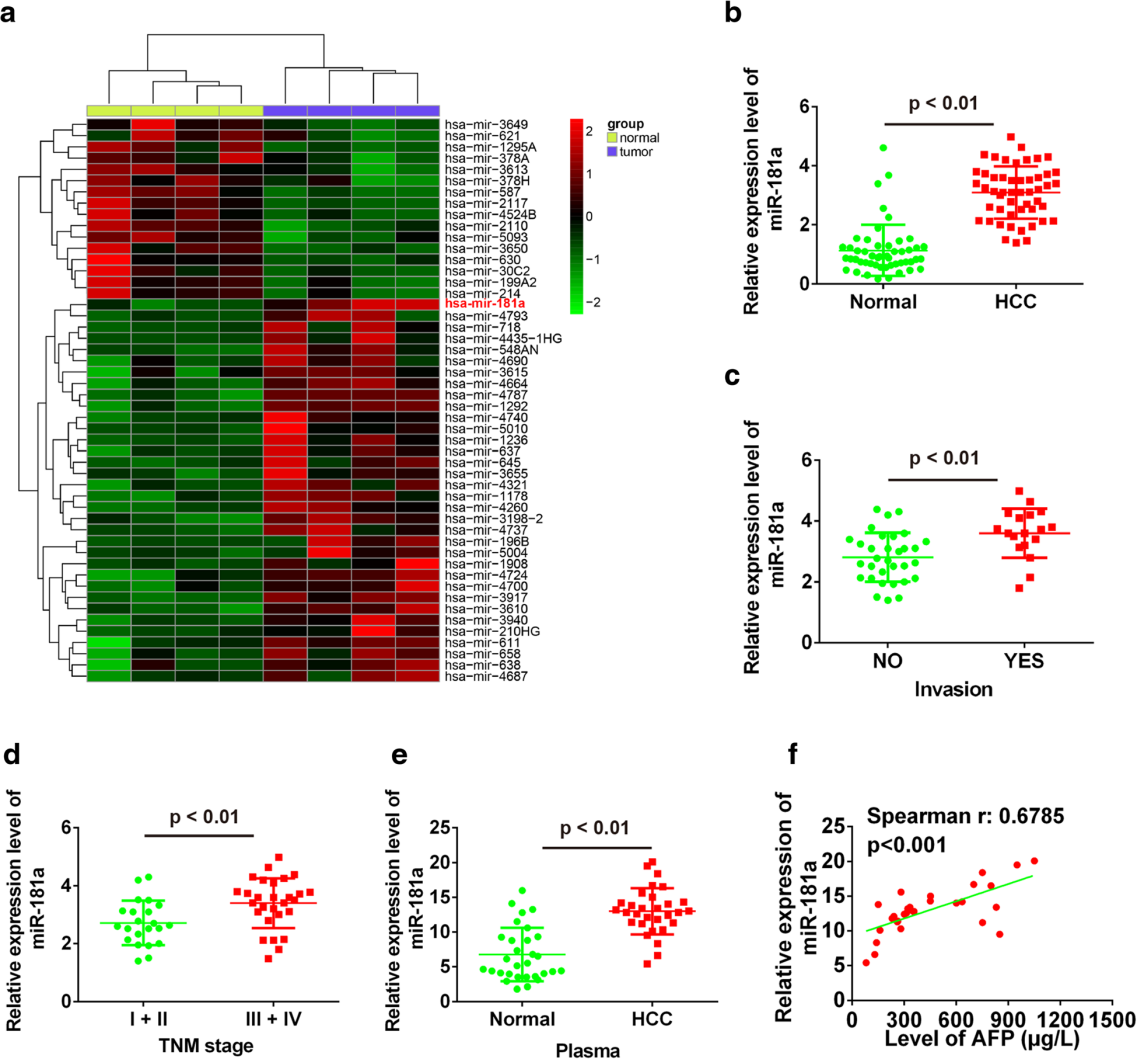
Expression levels of miR-181a in HCC tissues and plasma samples. **a** Partial miRNAs expression profiles of HCC tissues. **b** Relative expression level of miR-181a in HCC tissues and normal liver tissues. **c** Relative expression level of miR-181a in HCC metastatic and non-metastatic tissues. **d** Relative expression level of miR-181a in different TNM stage. **e** Relative expression level of miR-181a in serum of HCC patients and normal. **f** The positive correlation between AFP and miR-181a levels in the plasma of HCC patients. HCC, hepatocellular carcinoma

To explore whether and how miRNAs play key roles in HCC, miRNA data were downloaded from the database and uploaded to GEO (http://www.ncbi.nlm.nih.gov/geo/query/acc.cgi?acc=GSE77314) to screen differentially expressed genes. Results showed 16 miRNAs were down-regulated and 34 miRNAs were up-regulated (Fig. 1a). Among the 34 up-regulated miRNAs, the degree of up-regulation of miR-181a was the most marked. To explore the influence of miR-181a on HCC, 55 HCC tissue samples and the adjacent normal tissues were selected. The relative mRNA level of miR-181a in them was determined and compared. Results showed the level of miR-181a in HCC tissues was significantly higher than that in the adjacent tissues (Fig. 1b). To study the relationship between miR-181a and tumor metastasis, we studied the level of miR-181a in the invasive and normal tissues. Results showed the miR-181a expression levels in metastatic HCC tissues were dramatically higher than those in non-metastatic HCC tissues (Fig. 1c). Moreover, the levels of miR-181a in HCC tissues at different stages were also determined by qRT-PCR. Results showed the level of miR-181a in stage III and IV was markedly higher than that during stage I and II (Fig. 1d). It indicated miR-181a may be closely related to the TNM stage of HCC. Then the level of miR-181a in serum of HCC patients was determined and compared with normal levels. The results showed that the miR-181a level in HCC serum was significantly higher than normal (Fig. 1e). Moreover, the level of miR-181a was positively related with alpha feto protein (AFP) (Fig. 1f). AFP is a fetal glycoprotein produced by the yolk sac and fetal liver. It was reported to have a role in diagnosing and managing HCC [13]. These results indicated miR-181a is a potential biomarker to aid in HCC detection.

### Knockdown of miR-181a inhibited the proliferation, mobility, and invasion of HCC cells

**Fig. 2:**
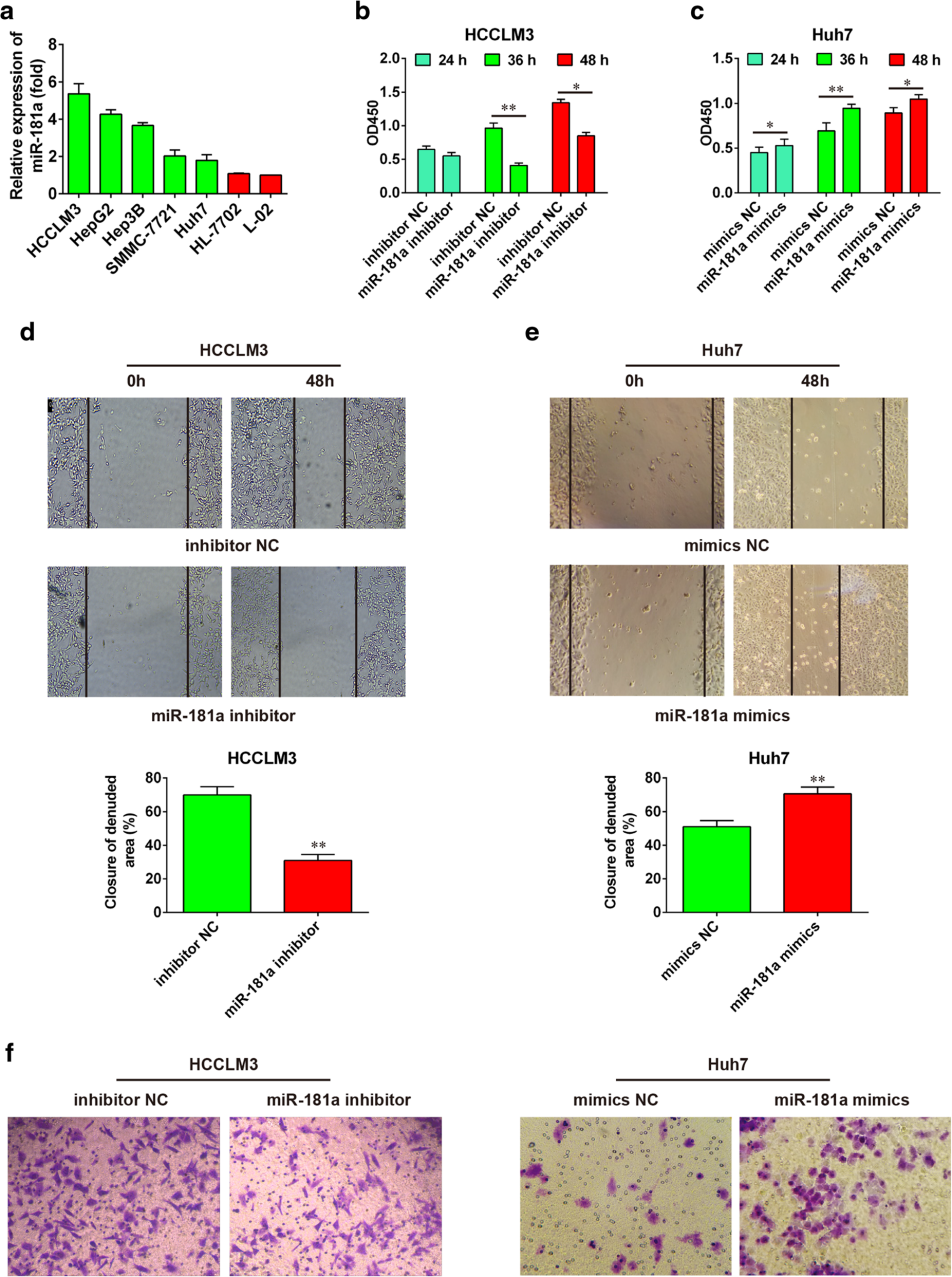
MiR-181a regulated the proliferation and invasion of HCC cells. **a** Relative expression level of miR-181a in HCC cell lines with high or low metastatic capacity and normal liver cells. **b** The proliferation of HCCLM3 cells with strong metastatic capacity was reduced after knockdown of miR-181a. **c** The proliferation of Huh7 cells with weak metastatic capacity was increased after the overexpression of miR-181a. **d** and **e** Representative micrographs of wound healing assay of HCCLM3 and Huh7 cells stably expressing miR-181a. **f** The effects of miR-181a overexpression or knockdown on invasion of HCCLM3 or Huh7 cells were analyzed using Transwell assays. HCC, hepatocellular carcinoma. All values are mean ± SD. * vs inhibitor NC, *p* < 0.05. ** vs inhibitor NC, *p* < 0.01

**Fig. 3:**
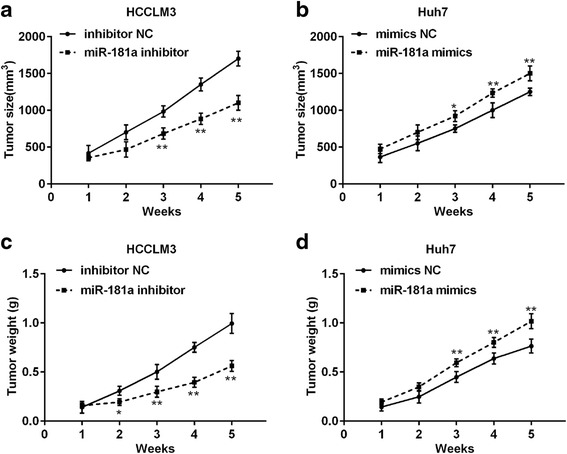
MiR-181a increased tumor size and weight. **a** Tumor size after transfection with inhibitor NC or miR-181a inhibitor. **b** Tumor size after transfection with mimics NC or miR-181a mimics. **c** Tumor weight after transfection with inhibitor NC or miR-181a inhibitor. **d** Tumor weight after transfection with mimics NC or miR-181a mimics. ** vs inhibitor NC or mimics NC, *p* < 0.01. *vs inhibitor NC or mimics NC, *p* < 0.05

To investigate the role of miR-181a in the development and progression of HCC, we measured miR-181a expression levels in various HCC cell lines (HCCLM3, HepG2, Hep3B, SMMC-7721, and Huh7) and normal liver cell lines (HL-7702 and L-02). Among these cell lines, HCCLM3 is a poorly differentiated HCC cell line with strong metastatic potential. Huh7 is a well-differentiated HCC cell line with weak metastatic ability. The results showed that in HCC cell lines, the relative expression of miR-181a in HCCLM3 cells was the highest, while the value in Huh7 cells was the lowest (Fig. 2a). HCCLM3 and Huh7cells were selected for further study. MiR-181a inhibitor was used to reduce the level of miR-181a in HCCLM3 cells, and miR-181a level was increased by miR-181a mimics in Huh7 cells. The transfection effect is illustrated in Additional file 3: Figure S1. As shown, the miR-181a inhibitor significantly decreased miR-181a level in HCCLM3 cells compared with the inhibitor NC group, whereas miR-181a mimics observably increased miR-181a level in Huh7 cells compared with the NC mimic group. When the proliferation capacity of HCCLM3 cells after transfection with miR-181a inhibitor for 24, 36, and 48 h was determined, cell growth decreased significantly after transfection with miR-181a inhibitor for 36 and 48 h (Fig. 2b). To verify the relationship between miR-181a and tumor growth, the level of miR-181a in Huh7 cells was increased by transfection with miR-181a mimics and cell viability was determined after 24, 36, and 48 h. Results showed the cell vitality increased after transfection with miR-181a mimics (Fig. 2c). Moreover, a wound-healing assay showed that knockdown of miR-181a drastically suppressed the mobility of HCCLM3 cells compared with control cells, whereas miR-181a overexpression promoted the mobility of Huh7 cells (Fig. 2d and e). Transwell assays demonstrated that knockdown of miR-181a significantly inhibited the invasion of HCCLM3 cells compared with that of the control cells, while miR-181a overexpression promoted the invasion of Huh7cells (Fig. 2f). Results of tumor size showed knockdown of miR-181a significantly reduced the tumor size compared with the control, while overexpression of miR-181a increased tumor size (Fig. 3a and b). Moreover, results of tumor weight also showed that knockdown of miR-181a dramatically reduced the tumor weight compared with the control, while overexpression of miR-181a increased tumor weight (Fig. 3c and d).

### MiR-181a regulated EMT by PI3K/AKT signaling pathway

**Fig. 4:**
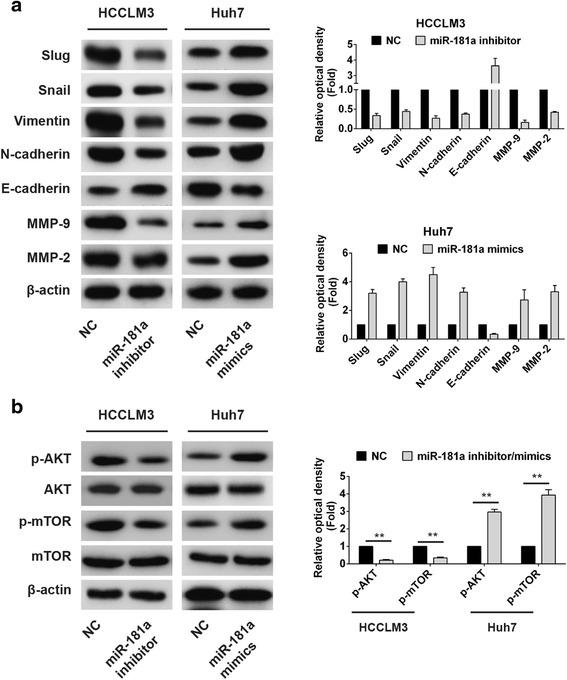
MiR-181a promoted HCC cell invasion and EMT by activating AKT signaling. **a** The protein expression of EMT related genes (Snail, Slug, N-cadherin, Vimentin and E-cadherin), MMP-2, and MMP-9. **b** Western blot analysis of phosphorylated (active) AKT and mTOR in HCC cells transfected with miR-181a mimics or inhibitor. HCC, hepatocellular carcinoma. EMT, epithelial-to-mesenchymal transition. All values are mean ± SD. ** vs NC group, *p* < 0.01

E-cadherin is a marker of epithelial cells, and MMP-2 and MMP-9 are markers of mesenchymal cells. Their changes correlated with EMT [14]. We examined the expression of EMT markers (Snail, Slug, N-cadherin, Vimentin, and E-cadherin), MMP-2, and MMP-9 in HCCLM3 and Huh7 cells. The results showed the expression of Snail, Slug, N-cadherin, Vimentin, MMP-2, and MMP-9 in HCCLM3 cells decreased after the knockdown of miR-181a, while the expression of E-cadherin increased. In Huh7 cells, we found increased expression of Snail, Slug, N-cadherin, Vimentin, MMP-2, and MMP-9 but E-cadherin expression decreased after transfection with miR-181a mimics (Fig. 4a). Many studies have shown that GSK3b is a target of PI3K/Akt and can regulate MMPs [15]. We examined the effect of miR-181a on AKT signaling pathway and found that knockdown of miR-181a significantly decreased the expression of phosphorylated AKT and mTOR in HCCLM3 cells, whereas overexpression of miR-181a significantly increased the expression of phosphorylated AKT and mTOR in Huh7 cells (Fig. 4b).

### PTEN is a direct target of miR-181a in HCC

**Fig. 5:**
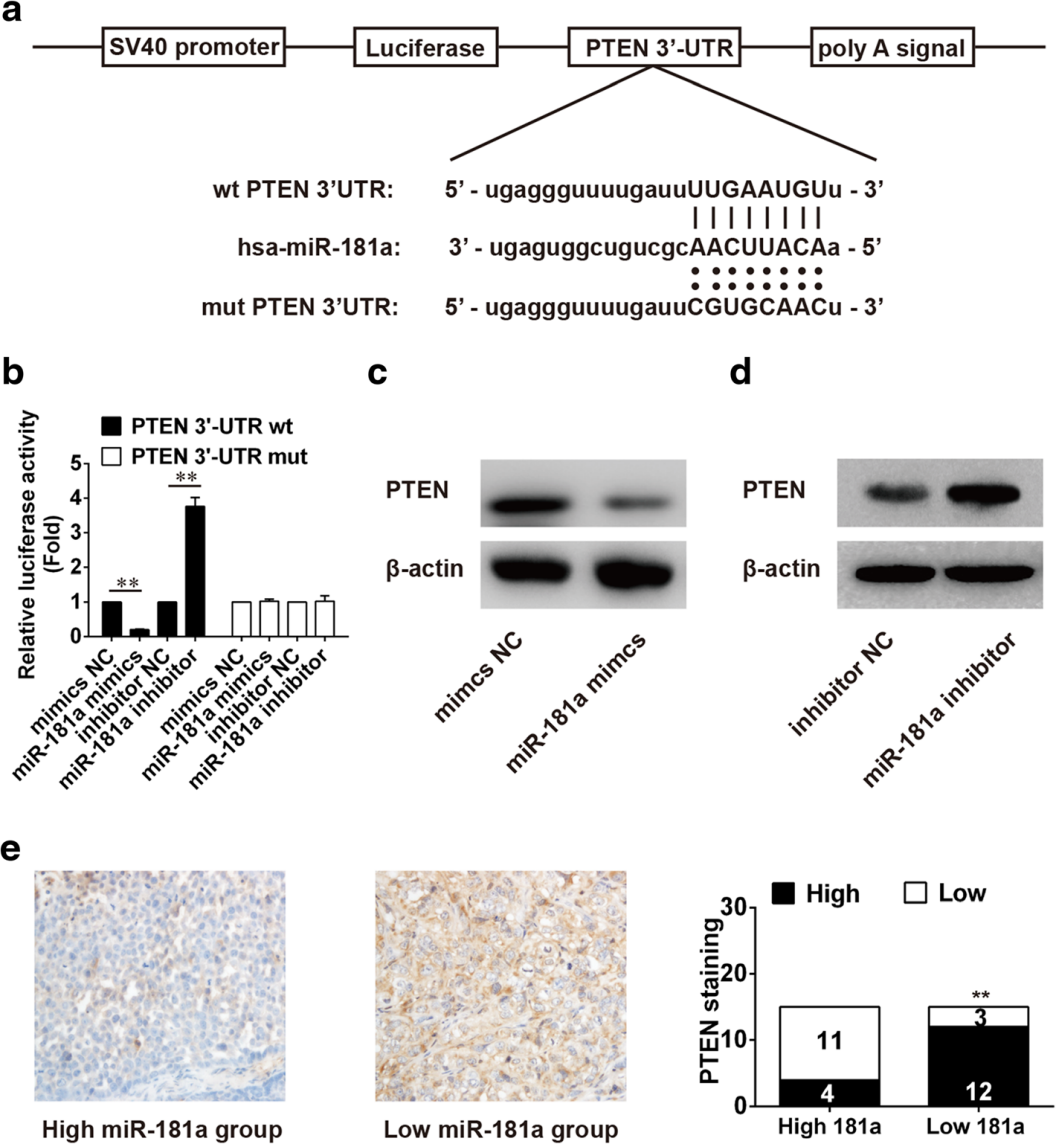
PTEN is the target gene of miR-181a. **a** Diagram of the miR-181a putative binding sites and corresponding mutant sites in the 3′-UTR of PTEN. **b** Effects of miR-181a on the expression of PTEN 3′-UTR-containing reporter genes. ** vs inhibitor NC or mimics NC, *p* < 0.01. **c** and **d** Protein expression of PTEN in HCC cells after transfection with miR-181a mimic or inhibitor. **e** The expression of PTEN in clinical samples with high or low level of miR-181a. mut, mutant; HCC, hepatocellular carcinoma. ** vs high 181a group, *p* < 0.01

By target gene prediction analysis, we found that there were binding sites between PTEN and microRNA. PTEN is a phosphatase with a sequence similar to that of the cytoskeletal protein tensin. Lee et al. [16] reported that in ovarian cancer, reduction of PTEN can activate the PI3K/Akt pathway. Moreover, treatment of PTEN-positive T-ALL cells can down-regulate constitutive phosphorylation of Akt in many leukemia/lymphoma cell lines [17]. Therefore, we selected PTEN for further study. As Fig. 5a showed, PTEN may be a target gene of miR-181a (Fig. 5a). The luciferase assay showed the miR-181a mimic inhibited the luciferase activity of PTEN 3′-UTR wt (wild type), but had no influence on the luciferase activity of PTEN 3′-UTR mut (mutant). MiR-181a inhibitor promoted the luciferase activity of PTEN 3′-UTR wt while it had no influence on the luciferase activity of PTEN 3′-UTR mut (Fig. 5b). Moreover, transfection with miR-181a mimics could significantly decrease PTEN expression, although transfection with miR-181a inhibitor significantly increased PTEN expression (Fig. 5c and d). The results of immunohistochemistry studies (Fig. 5e) showed PTEN staining decreased when the expression of miR-181a was low. All these results indicated PTEN was a target gene of miR-181a.

### Reciprocal repression between XIST and miR-181a in HCC cells

**Fig. 6:**
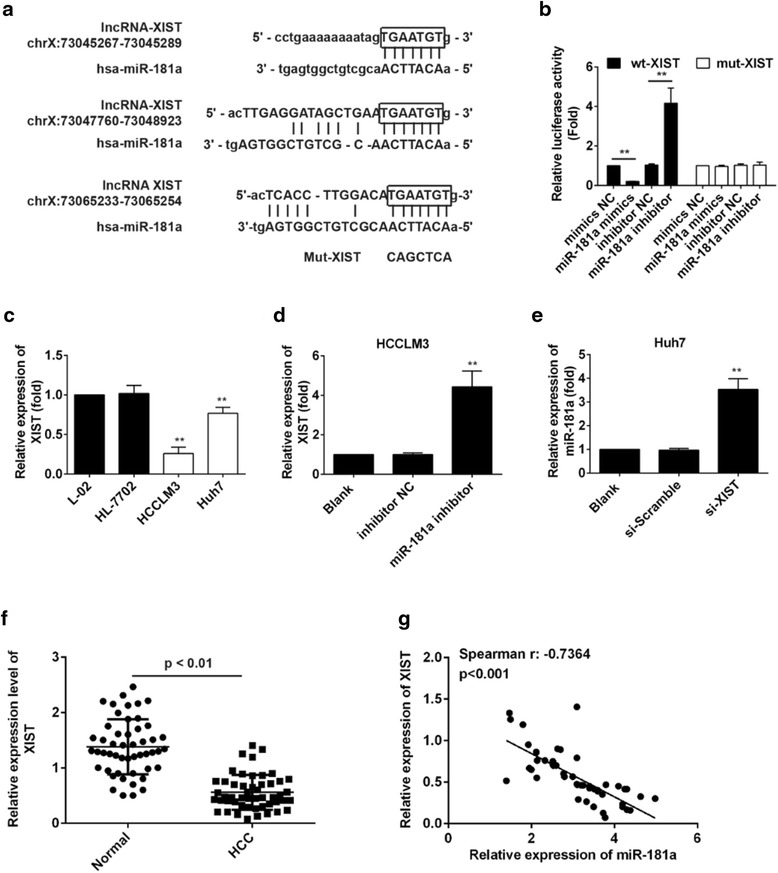
miR-181a is regulated by lncRNA XIST. **a** Diagram of the three miR-181a putative binding sites and corresponding mutant sites in XIST mRNA sequences. **b** Effects of miR-181a on the expression of reporter genes containing wt-XIST or mut-XIST sequences. ** vs Blank, *p* < 0.01. **c** Relative expression of XIST in HCC cells and the normal liver cells. ** vs L-02, *p* < 0.01. **d** Relative expression of XIST in HCCLM3 cells after transfection with miR-181a inhibitor. ** vs Blank, *p* < 0.01. **e** Relative expression of miR-181a in Huh7 cells after transfection with si-XIST or si-Scramble. ** vs Blank, *p* < 0.01. **f** Relative expression of miR-181a in HCC clinical samples and the normal. **g** The relationship between relative expression of XIST and miR-181a. lncRNA, long non-coding RNA; mut, mutant; wt, wild-type; HCC, hepatocellular carcinoma

Recent studies showed that microRNA-92b can promote HCC progression by targeting Smad7, mediated by lncRNA XIST [18]. So we explored whether the regulation on PTEN by miR-181a is regulated by lncRNA. Using bioinformatics analysis, we found binding sites between miR-181a and lncRNA XIST, and thus carried out the follow-up study. To explore the interaction between XIST and miR-181a, we constructed three reporter plasmids separately containing one predicted miR-181a binding site on the mRNA of XIST, and three corresponding reporter plasmids with mutant miR-181a binding sites (Fig. 6a). The results of the three binding sites were same (data not shown) so one binding site was selected for further study. Luciferase reporter gene assay showed that miR-181a could significantly inhibit the reporter activities of wt-XIST but not mut-XIST (Fig. 6b). We subsequently detected the expression of XIST in normal liver cells L-02 and HL-7702, and in HCC cells HCCLM3 and Huh7. The results showed the level of XIST in normal liver cells was markedly higher than that in HCC cells. Moreover, the level in Huh7 cells was significantly higher than that in HCCLM3 cells (Fig. 6c). Transfection was used to change the level of XIST in HCC cells. The XIST level in Huh7 cells decreased significantly after transfection with si-XIST (Additional file 4: Figure S2A), whereas XIST level in HCCLM3 cells increased markedly after transfection with pcDNA-XIST (Additional file 4: Figure S2B). Figure 6d shows that the level of XIST in the miR-181a inhibitor group increased significantly compared with both the blank and the inhibitor NC group. The relative expression of miR-181a increased markedly in si-XIST group compared with the blank group (Fig. 6e). Then, we used RT-PCR to determine the relative expression of XIST and the results showed the level in HCC tissues was lower than that in normal tissues (Fig. 6f). The relationship between the relative expression of XIST and miR-181a was determined, and the results showed that the level of XIST was inversely proportional to the miR-181a level (Fig. 6g).

### LncRNA XIST regulated HCC via miR-181a

**Fig. 7:**
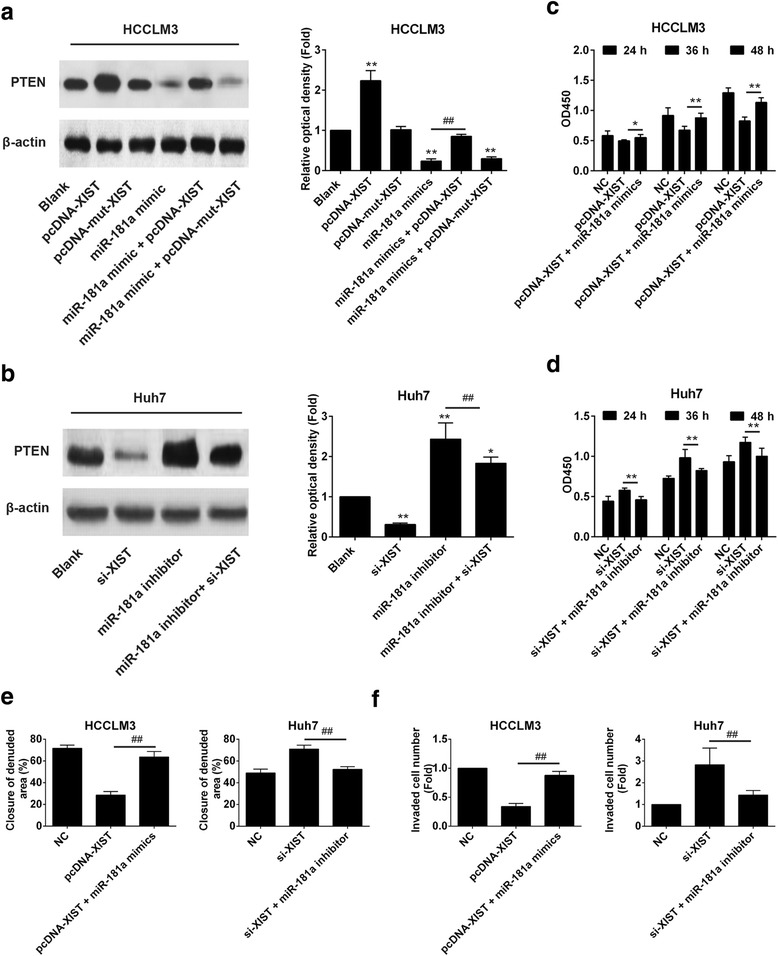
LncRNA XIST regulates HCC via miR-181a. **a** and **b** The regulation of XIST on PTEN was mediated by miR-181a. * vs Blank, *p* < 0.05. ** vs Blank, *p* < 0.01. ^##^ vs miR-181a mimics or miR-181a inhibitor group, *p* < 0.01. **c** and **d** The regulation of XIST on HCC proliferation was mediated by miR-181a. ** vs si-XIST or pcDNA-XIST group, *p* < 0.01. ^##^ vs miR-181a mimics or miR-181a inhibitor group, *p* < 0.01. **e** and **f** XIST inhibited the migration and invasion of HCCLM3 cells, while the transfection of miR-181a mimics abolished the inhibition. lncRNA, long non-coding RNA; HCC, hepatocellular carcinoma. ^##^ vs si-XIST or pcDNA-XIST group, *p* < 0.01

To explore the relationship between lncRNA XIST and miR-181a, the protein expression values of PTEN in HCCLM3 cells after transfection with pcDNA-XIST, pcDNA-mut-XIST, miR-181a mimic, miR-181a mimic + pcDNA-XIST, and miR-181a mimic + pcDNA-mut-XIST were determined by western blotting. The results showed that the expression value of PTEN in the miR-181a mimic group was decreased and in the pcDNA-XIST group it was increased compared with the blank group. However, the inhibition of miR-181a mimic on PTEN was abolished by pcDNA-XIST, while further use of pcDNA-mut-XIST had no influence on the inhibition effect of miR-181a mimic on PTEN (Fig. 7a). Moreover, the protein expression value of PTEN was decreased by si-XIST and increased by miR-181a inhibitor, and the promotion of miR-181a inhibitor on PTEN expression was abolished by si-XIST (Fig. 7b). Results of a CCK-8 assay showed pcDNA-XIST reduced the growth of HCCLM3 cells, while pcDNA-XIST + miR-181a mimic increased the proliferation of HCCLM3 cells. Results of the CCK-8 assay showed that the proliferation of cells with pcDNA-XIST was significantly reduced compared with that of control cells. In contrast, overexpression of miR-181a in HCCLM3 cells could abolish the weakening effects of pcDNA-XIST (Fig. 7c). The proliferation of cells treated with siXIST was significantly enhanced compared with that of control cells, whereas knockdown of miR-181a in Huh7 cells could abolish the proliferative effects of siXIST (Fig. 7d). The wound healing assay and Transwell assay showed that migration and invasion of cells treated with siXIST was significantly enhanced compared with that of the control. Meanwhile, knockdown of miR-181a in Huh7 cells abolished the enhancing effects of siXIST on cell migration and invasion. Moreover, both migration and invasion of HCCLM3 cells with pcDNA-XIST were significantly reduced compared with that of control cells. Additionally, overexpression of miR-181a in HCCLM3 cells abolished the effects of pcDNA-XIST to reduce cell migration and invasion (Fig. 7e and f). XIST levels were significantly decreased in HCC and associated with histological grade and tumor-node-metastasis stage [19]. These results indicated that LncRNA XIST and miR-181a could directly interact with and repress each other, and XIST could inhibit HCC cell proliferation and metastasis by targeting miR-181a.

## Discussion

Hepatocellular carcinoma (HCC) is a serious disease [20] that is complex and heterogeneous [21]. Difficult complications of HCC include metastatic lesions and postsurgical recurrence [22]. Tumor metastasis seriously affects the prognosis of HCC. Recently, microRNAs have been discovered to have a role in metastasis and have been used as cancer-related biomarkers [23]. They are involved in regulating tumor metastasis via inhibition of numerous of target genes [24]. The study of Huang et al. showed that miR-373 and miR-520c could promote breast cancer invasion and metastasis and are regarded as metastasis-promoting miRNAs [25]. MicroRNA-34a inhibits prostate cancer metastases by directly repressing CD44 [26]. Therefore, we expected to identify microRNAs that regulate HCC metastasis by analyzing microRNA expression profiles. The expression profiles showed 16 miRNAs were down-regulated and 34 miRNAs were up-regulated.

MicroRNA-181a can promote gastric cancer by negatively regulating tumor suppressor KLF6 [27]. It can regulate EMT in ovarian cancer, cause hepatitis B virus-related HCC, and is related to EpCAM^+^ HCC cell quantity and tumor initiating ability [28–30]. Among the 34 up-regulated miRNAs identified by analyzing microRNA expression profiles, we found up-regulation of miR-181a was the highest. Moreover, the level of miR-181a in HCC tissues was significantly higher than that in the normal liver tissues. Hepatitis B virus (HBV) is involved in the initiation and progression of HCC, the expression of miR-181a was strongly up-regulated in HBV-expressing cells or HCC cells [29, 31, 32]. Liu et al. used miRNA microarrays and northern blotting analyses to compare the expression profile of cellular miRNAs of a stable HBV-expressing cell line (HepG2.2.15) and its parent cell line HepG2, and the results showed that the expression of miR-181a, miR-181b, miR-200b, and miR-146a were all up-regulated in HepG2.2.15 cells [33]. Zhuo et al. reported up-regulation of miR-27b, miR-181a, miR-146b-5p, miR-181d, and miR-146a expression using real-time RT-PCR in five different drug-resistant HCC cell sublines [33]. Moreover, the expression of miR-181a in HCC liver tissues was also up-regulated compared with the normal cells [34–36]. However, Elhelw et al. reported that no difference in miR-181a expression was observed in liver tissues and peripheral blood mononuclear cell (PBMCs) of patients compared with controls [37]. Korhan et al. reported miR-181a-5p is down-regulated in hepatocellular carcinoma and suppresses motility, invasion and branching-morphogenesis by directly targeting c-Met [38]. This may be because in the study by Elhelw, the tissues collected from HCV-infected patients may have been the adjacent tissues, which led to the result that miR-181a expression in the liver tissues of patients was not different from the controls. Our study showed miR-181a level in HCC tissues was significantly higher than the normal liver tissues. That was because some studies have shown that miR-181a is down-regulated and others have reported the up-regulation of miR-181a during HCC. Combining this data with the above research, we confirmed that up-regulated miR-181a plays a key role in regulating HCC.

Up-regulated miR-181a was reported to promote tumor metastasis. Our study showed the expression level of miR-181a in metastatic HCC tissues was significantly higher than in non-metastatic HCC tissues. Moreover, the level of miR-181a in stage III and IV tumor was markedly higher than that in I and II stage. The value in serum of HCC patients was also much higher than normal, and the expression of miR-181a was positively related with AFP level. AFP was reported to be a reliable serum marker for HCC [39]. Therefore, we concluded that miR-181a has the potential to be used as a biomarker for HCC. We also determined the expression levels of miR-181a in HCC cell lines with strong or weak metastasis ability. Results showed the miR-181a expression in HCC cell lines with strong metastatic tendencies was significantly higher than in the HCC cell lines with low metastatic tendencies. Combining with the result that miR-181a level in metastatic HCC tissues was significantly higher than in non-metastatic HCC tissues, we speculated that miR-181a was closely related to HCC metastasis. Furthermore, we found that knockdown of miR-181a inhibited the cell proliferation, scratch repair and cell invasion of HCC cells, whereas overexpression of miR-181a promoted the cell proliferation, scratch repair and cell invasion. All those indicated miR-181a expression played a key role in HCC invasion and metastasis.

EMT is a key factor to embryonic development and tumor metastasis [7]. Our study showed that the expression levels of EMT-related protein Slug, Snail, Vimentin, and N-cadherin in HCC cells decreased markedly after the knockdown of miR-181a, but the expression of E-cadherin increased. We concluded that the knockdown of miR-181a induced the production of EMT, which plays a key role in tumor metastasis. The levels of MMP2 and MMP9 decreased with the reduction of FAP level. MMP2 and MMP9 belong to MMP family, which have important roles in tumor cell invasion and metastasis [40]. Many studies have shown that there are many signaling pathways that take part in mediating EMT [41]. In this study, we tested multiple signaling pathways that were related to EMT, and interestingly we found that the PI3K/AKT/mTOR pathway was regulated by microRNA. The PI3K pathway can regulate survival signals, prevent the apoptosis of HCC cells, and promote oncogenic transformation [42–44]. PTEN can be used to suppress tumors and inhibit the activation of PI3K/AKT signaling pathway [45]. The loss of PTEN can result in the activation of AKT kinases, which play key roles in cell growth, proliferation and invasion [46]. Here, we identified PTEN as a direct and functional target of miR-181a. The study showed that overexpression of miR-181a inhibited the expression of PTEN, and knockdown of miR-181a promoted its expression. We concluded that up-regulated miR-181a decreased the expression of PTEN, and the loss of the PTEN activated PI3K/AKT signaling pathway then promoted the HCC metastasis in HCC.

Recently, more and more studies have suggested that microRNAs are regulated by lncRNA. We hypothesized that the role of miR-181a in promoting HCC metastasis is regulated by lncRNA. The software analysis found that miR-181a has multiple binding sites with the lncRNA XIST. XIST was reported to have key roles in many tumors including testicular germ cell tumors and ovarian cancer [47]. Our results showed for the first time that knockdown of XIST up-regulated the level of miR-181a and knockdown of miR-181a promoted the expression of XIST. Moreover, our study showed overexpression of miR-181a reduced the expression of PTEN, while the further transfection with pcDNA-XIST abolished this effect. The proliferation, migration, and invasion of HCC cells after XIST overexpression were significantly weakened compared with those of control cells. PcDNA-XIST could reduce the migration and invasion of HCC cells. After transfection with miR-181a mimics, this effect was abolished. However, the transfection with miR-181a inhibitor abolished the increase effect of siXIST on HCC cell migration and invasion. We speculated that miR-181a and XIST are important in the development of HCC.

## Conclusions

Taken together, our study not only revealed the important role of XIST/miR-181a/PTEN signaling pathway in HCC pathogenesis, but also implied a potential role for both miR-181a and XIST in the clinical diagnosis and treatment of HCC.

## Additional files

Additional file 1: Table S1. Primers used for quantitative real-time PCR. (DOCX 15 kb)

Additional file 2: Table S2. Sequences including the siRNA and the scramble sequence used in transfection assay. (DOCX 15 kb)

Additional file 3: Figure S1. (A) Relative expression of XIST in Huh7 cells after transfection with si-XIST or si-Scramble. ** vs Blank, *p* < 0.01. (B) Relative expression of XIST in HCCLM3 cells after transfection with pcDNA-XIST or pcDNA-Scramble. ^##^ vs Blank, *p* < 0.01. (TIFF 164 kb)

Additional file 4: Figure S2. (A) The efficiency of miR-181a inhibitor transfected in HCCLM3 cells was evaluated by qRT-PCR. ** vs inhibitor NC, *p* < 0.01. (B) The efficiency of miR-181a mimics transfected in Huh7 cells was evaluated by qRT-PCR. ^##^ vs mimics, *p* < 0.01. (TIFF 118 kb)

## Abbreviations

AJCC: American Joint Committee on Cancer
BSA: bovine serum albumin
EMT: Epithelial-to-mesenchymal transition
ERGIC3: endoplasmic reticulum-golgi intermediate compartment protein 3
FBS: fetal bovine serum
GAPDH: glyceraldehyde-3-phosphate dehydrogenase
HCC: Hepatocellular carcinoma
MiRNA: microRNA
RIPA: radioimmunoprecipitation assay
TBST: Tris-buffered saline Tween-20
TNM: tumor node- metastasis
