# Supplementary material for: Long non-coding RNA XIST regulates PTEN expression by sponging miR-181a and promotes hepatocellular carcinoma progression
Source: BMC Cancer. 2017 Apr 7;17:248. doi: 10.1186/s12885-017-3216-6 (PMC5383949; doi:10.1186/s12885-017-3216-6)
Supplement: Supplementary file 1 — Primers used for quantitative real-time PCR. (DOCX 15 kb) [file 12885_2017_3216_MOESM1_ESM.docx]

**Table S1** Sequences including the siRNA and the scramble sequence used in transfection assay

| **Names** | **Sequences (5’-3’)** |
| --- | --- |
| si-XIST-1 | GGCCTGTTATGTGTGTGATTATATT |
| si-XIST-1 scramble | GGCATTGGTGTGTGTATTATTCATT |
| si-XIST-2 | CACAACCATGCATCTTGGAAATTTA |
| si-XIST-2 scramble | CACTACCTACGGTTCAAAGTAATTA |
| si-XIST-3 | GAGGCCTCTTTGCTTGGCTTGTCTA |
| si-XIST-3 scramble | GAGTCTCCGTTGGTTGTTCTCGCTA |
