# Supplementary material for: Long non-coding RNA XIST regulates PTEN expression by sponging miR-181a and promotes hepatocellular carcinoma progression
Source: BMC Cancer. 2017 Apr 7;17:248. doi: 10.1186/s12885-017-3216-6 (PMC5383949; doi:10.1186/s12885-017-3216-6)
Supplement: Supplementary file 2 — Sequences including the siRNA and the scramble sequence used in transfection assay. (DOCX 15 kb) [file 12885_2017_3216_MOESM2_ESM.docx]

**Table S2** Primers used for quantitative real-time PCR

| **Names** | **Primer sequences (5’-3’)** |
| --- | --- |
| miR-181a-5p RT | GTCGTATCCAGTGCAGGGTCCGAGGTATTCGCACTGGATACGACACTCAC |
| miR-181a-5p F | GAACATTCAACGCTGTCGGTGA |
| U6 F | CTCGCTTCGGCAGCACA |
| U6 R | AACGCTTCACGAATTTGCGT |
| XIST F | AGCTCCTCGGACAGCTGTAA |
| XIST R | CTCCAGATAGCTGGCAACC |
