# Supplementary figures and images for: Long non-coding RNA XIST regulates PTEN expression by sponging miR-181a and promotes hepatocellular carcinoma progression
Source: BMC Cancer. 2017 Apr 7;17:248. doi: 10.1186/s12885-017-3216-6 (PMC5383949; doi:10.1186/s12885-017-3216-6)

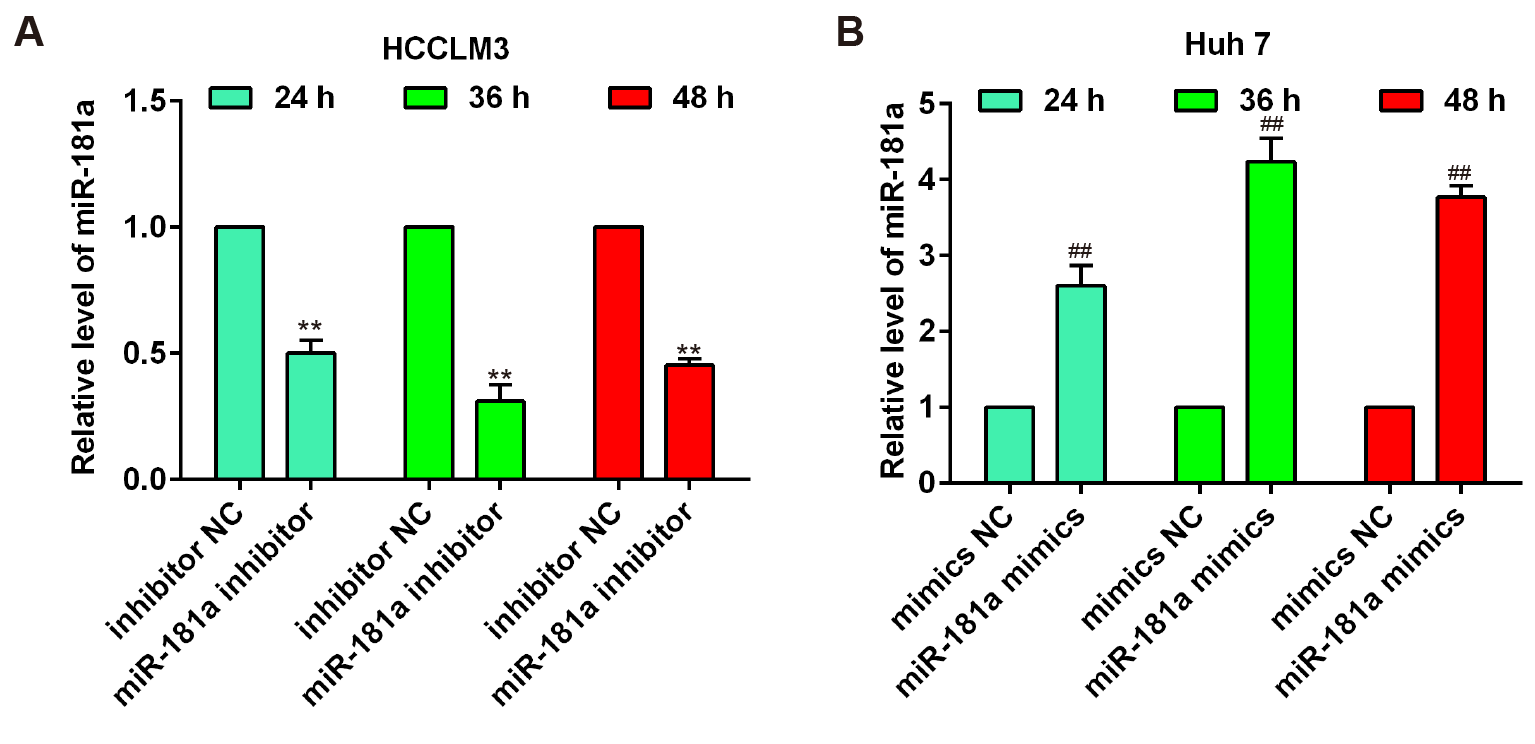

Supplement: Supplementary file 3 — (A) Relative expression of XIST in Huh7 cells after transfection with si-XIST or si-Scramble. ** vs Blank, p < 0.01. (B) Relative expression of XIST in HCCLM3 cells after transfection with pcDNA-XIST or pcDNA-Scramble. ## vs Blank, p < 0.01. (TIFF 164 kb) [file 12885_2017_3216_MOESM3_ESM.tif]

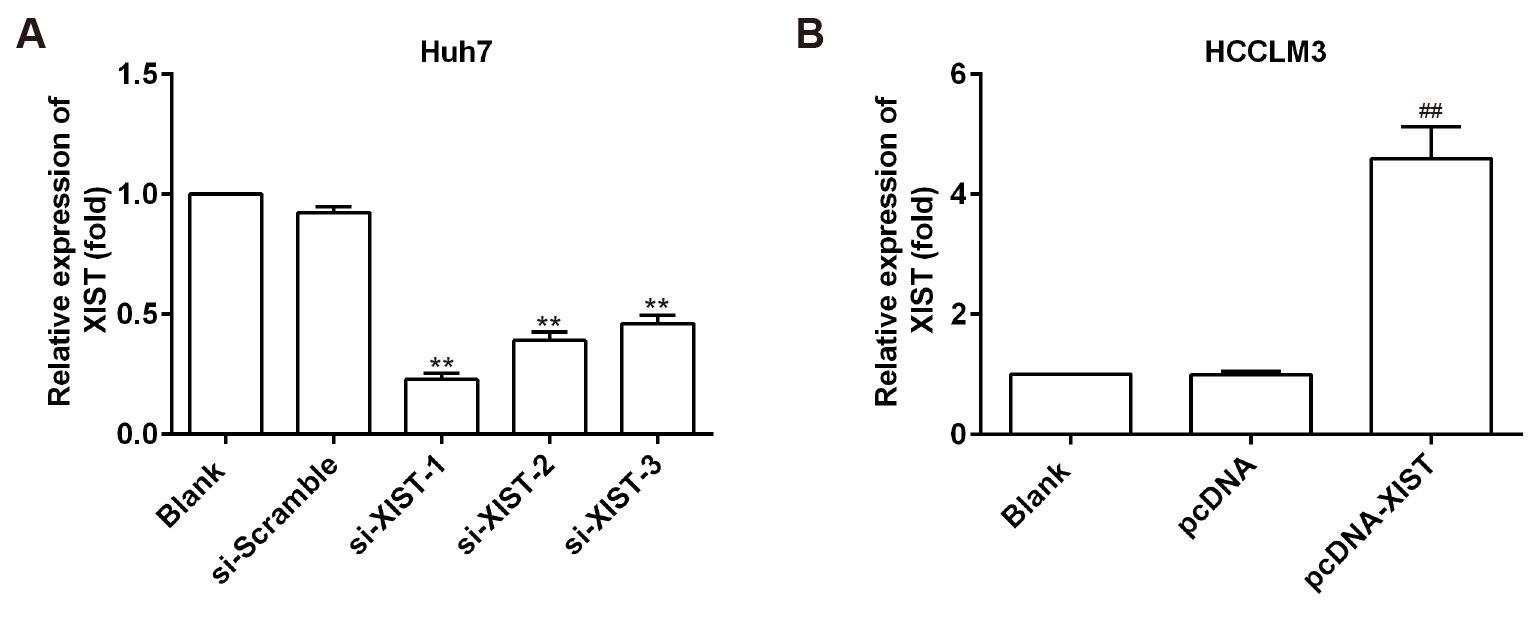

Supplement: Supplementary file 4 — (A) The efficiency of miR-181a inhibitor transfected in HCCLM3 cells was evaluated by qRT-PCR. ** vs inhibitor NC, p < 0.01. (B) The efficiency of miR-181a mimics transfected in Huh7 cells was evaluated by qRT-PCR. ## vs mimics, p < 0.01. (TIFF 118 kb) [file 12885_2017_3216_MOESM4_ESM.tif]
